# Recovering Evaluation of Narrow-Kerf Teeth of Mini Sash Gang Saws

**DOI:** 10.3390/ma14237459

**Published:** 2021-12-05

**Authors:** Kazimierz Antoni Orlowski, Daniel Chuchala, Tomasz Przybylinski, Stanislaw Legutko

**Affiliations:** 1 Faculty of Mechanical Engineering and Ship Technology, Gdańsk University of Technology, 11/12 Gabriela Narutowicza Street, 80-233 Gdańsk, Poland; daniel.chuchala@pg.edu.pl; 2 Institute of Fluid-Flow Machinery, Polish Academy of Sciences, 14 Fiszera Street, 80-231 Gdańsk, Poland; tprzybylinski@imp.gda.pl; 3 Faculty of Mechanical Engineering, Poznan University of Technology, 5 M. Skłodowska-Curie Square, 60-965 Poznan, Poland; stanislaw.legutko@put.poznan.pl

**Keywords:** narrow-kerf saw blade, stellite-tipped teeth, quality of sharpening, cutting edge radius, optical measurements

## Abstract

Sash gang saws with narrow-kerf saw blades are used in the production of glued laminate flooring elements in plants where dry technology is applied. This means that boards or friezes are sawn into top layer lamellae in dry conditions (moisture content of about 10–12%) from expensive wood species, often exotic. The object of this research was stellite-tipped teeth of narrow kerf saw blades sharpened under industrial conditions. A NIKON ECLIPSE Ti-S microscope equipped with a NIKON DS-Fi2 recording camera was used to take pictures of teeth, which were analysed in a graphical software to measure the radii of the main cutting edges. The high-quality images obtained were used to determine the values of the rounding radii of the cutting edges. It was noted that the quality of edges regenerated in industrial conditions, some of which had chipping, was lower than that of brand new saw blades.

## 1. Introduction

The accuracy of cutting, especially with narrow-kerf saw blades of low initial stiffness, depends mainly on the state of the cutting edges. It was found that the values of feed forces, occurring during wood cutting on sash gang saws, depend not only on cutting parameters but mainly on the stereometric features of the teeth and the accuracy of their manufacture [1]. High feed forces cause a reduction of initial stiffness values to operating stiffness values [2,3]. This phenomenon causes inaccuracies in sawing [4] because saw blades are sensitive not only to thrust forces but also to feed forces [1,2,3].

Piotrowski [5] described the effect of sharpening in workshop conditions on the accuracy of hobs, and proposed amendments to the rake face shaping technology allowing for improvement in the accuracy of the hobs. The application of numerical simulation to determine the ability of air used in the minimum quantity lubrication (MQL) method to clean grinding wheel active surfaces during the sharpening of hob cutters was proposed by Stachurski et al. [6]. The hob after the sharpening process can be identified with the use of the methods presented in [7]. The effect of sharpening on broaching efficiency was investigated by Jaworski et al. [8]. In [8], it was found that as the number of sharpenings increases, the mean value of the pull broach tooth wear on the clearance face, the magnitude of the standard deviation, and the coefficient of variance increase, which indicates a decrease in the reliability of the broach operation.

For many years, research on woodworking tools has focused on the issue of cutting edge wear [3,9,10]. The wear of a cutting edge depends not only on the cutting conditions but also on the structural and mechanical properties of the processed wood (e.g., anisotropy, density, annual rings, hardness), the defects it contains (knots), timber temperature, and moisture content. The wear process leads to bluntness of the cutting edge, which is often described by the cutting edge radius or the radial displacement the cutting edge [3]. Beer [10] proved that modifying a knife by creating a microphase on the cutting edge (14 μm) while beech wood veneer was peeled allowed for the tool life to be increased by almost 5 times. The cutting surfaces of sharpened hobs should be lapped to eliminate edge chipping, which, as a result, has a direct effect on the smoothness of the gear wheel’s involute [5].

The same goes when cutting metals, where it is recommended that new band saw blades are run-in in a controlled manner. This allows the blade life to be increased. To achieve this, the user should reduce the feed rate during the first cuts to 50% of the recommended feed rate. After cutting a section with a total surface of about 400 cm^2^, the feed rate can be gradually increased up to the optimum value [11].

Monitoring the degree of milling tool wear during machine tool processing can improve product quality and reduce production losses. The tool wear degree can be assessed according to the tool force data, vibration data, acoustic emission signal [12], temperature data [13], and other multi-sensor data, which were analysed with the Elman_Adaboost strong predictor [14]. Cutting forces and the acceleration of mechanical vibrations were also used to monitor the tool wear process during turning of hardened 100Cr6 bearing steel, with a hardness of 61 ± 1 HRC, with oxide ceramics (Al_2_O_3_ + TiN) as the tool material inserts. The presented neural network model revealed that the best correlation coefficient between tool wear, VB_C_, was obtained for the radial cutting forces F_p_ [15].

During the milling of a nickel–titanium (NiTi) shape memory alloy (SMA) with uncoated cutting tools with different nose radii under dry cutting conditions, the authors of [16] revealed that the wear parameter VB on the flank face depends mainly on the feed per tooth f_z_. The wear parameter VB on the flank face of the cutting tool (PVD coated inserts) while turning AISI 5140 steel under dry cutting conditions was dominated by cutting speed [17].

Furthermore, it is promising to determine the tool wear of the cutting tool during turning using a neural network based on infrared thermography [18]. The proposed model by Brili et al. [18] uses infrared thermography, computer vision, and deep learning and allows a 96% accuracy of assessment.

Investigations of the effect of manufacturing processes on the cutting edge quality and wear behaviour of end mills were presented in the work by Denkena et al. [19]. Moreover, current technology and production processes are not able to manufacture a cutting tool with the required accuracy and quality before inserting PVD coatings. For this reason, cutting edge preparation is inevitable and necessary [20].

The initial cutting edge radius after grinding for woodworking tools is commonly in the range of 10–15 μm [3,21]. Wasielewski and Orlowski [22] presented the automatic vision teeth controller system WKOPTar, developed at the Gdańsk University of Technology. This device only allows for the assessment of the cutting edges of the circular saw blades by macro measurements. Sandak et al. [23] and Palubicki et al. [24] created a low-cost device (ToolScan scanner) for virtually reproducing the cutting edge form and for supplying information about its 3D micro geometry. The latter scanner seems to be more universal; nevertheless, it does not work in the automatic manner.

At this point, it can be argued that the difference between the concept of sharpness and dullness (bluntness) becomes debatable. St. Thomas Aquinas [25] raised an interesting metaphysical question: “How many angels can dance on the head of a pin?”. There is no answer to that. Thus, the importance of the sharpness concept is rather relative to a particular cutting operation.

Knowing the value of the radius of the rounded cutting edge is important when determining the energy effects of the wood cutting process, since its value affects the specific cutting resistance [3,21,26].

The cutting forces during sawing can be used as a basis for determining raw material properties such as shear yield stresses in the cutting zone and fracture toughness [27,28]. Nevertheless, a prerequisite is the sharpness of the cutting blades, otherwise the fracture toughness results may be overestimated [29,30]. Childs [31] has shown that the value of the intercept from which fracture toughness could be determined depends on the value of the cutting edge radius.

**Figure 1:**
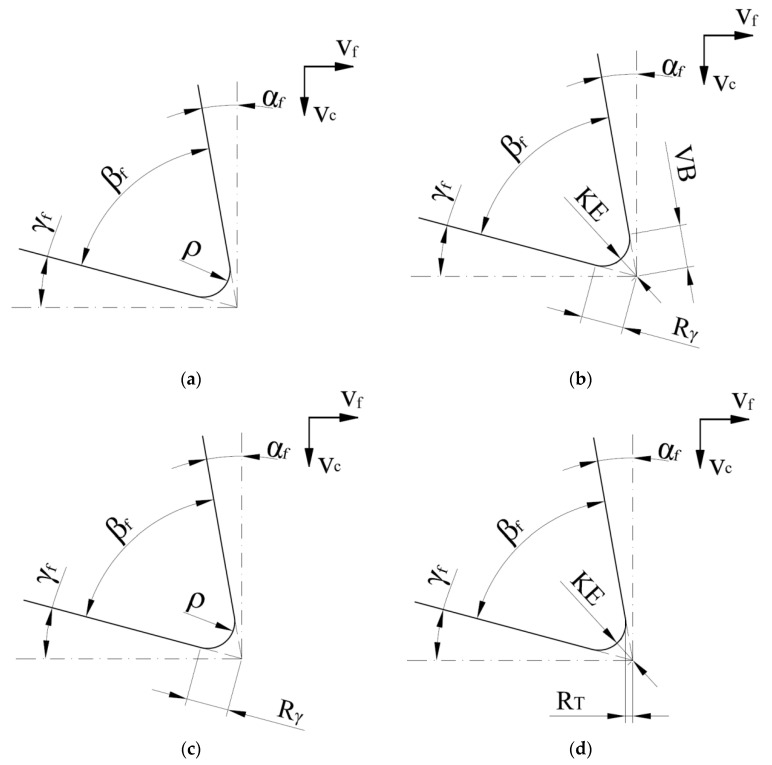
The blunt cutting edge parameters: (**a**) radius of the cutting edge ρ; (**b**) radial displacement of the tool corner KE, recessions on the flank VB and rake face Rγ; (**c**) radius of the cutting edge ρ and recession of the rake face Rγ; and (**d**) radial displacement of the tool corner KE and recession in tangential direction R_T_; Other symbols: γ_f_—tool side rake angle; α_f_—tool side clearance angle; β_f_—tool side wedge angle; v_c_—cutting speed; v_f_—feed speed.

In the case of metalworking tools, both the tool geometry and the tool wear can be described unambiguously [32], which unfortunately cannot be said for many woodworking tools. In Figure 1 there are some examples of the cutting edge wear descriptions in which the state of the cutting edge is presented in the simplest way by the radius of the cutting edge ρ (Figure 1a) [3,33], a radial displacement of the tool corner KE together with recessions on the flank VB and the rake face Rγ (Figure 1b) [34], the radius of the cutting edge ρ and recession of the rake face Rγ (Figure 1c) [35], and the radial displacement of the tool corner KE and recession in tangential direction R_T_ [36].

In the case of saw blades for woodworking, worn teeth made of stellite or cemented carbide are generally regenerated a few times by grinding. There is also a known Japanese solution in the form of replaceable special inserts made of stellite or high-speed steel for individual teeth of the bandsaw blade [37]. Nevertheless, this solution has not been widely used in European industry. Okai et al. [37] proved that silica accumulation species could have a significant effect on the tool wear of high-speed steels. Stellite inserts have the lowest cutting tool edge recession when machining wood samples of Oil palm (*Elais guineensis*). On the other hand, they were characterized by the largest recession when machining Afina (*Strombosia glaucescens*). In their experiments, the authors observed types of wear as presented in Figure 1b,c.

The purpose of this study was to determine the quality of the regeneration of mini sash gang saws by measuring the radii of the cutting edges. The regenerated saw blade was compared to the quality of a brand new narrow kerf saw blade.

## 2. Materials and Methods

The object of this research is stellite-tipped teeth of narrow-kerf saw blades: one by Wintersteiger (f. Wintersteiger AG, Ried, Austria) [38], sharpened under industrial conditions, and the second a brand new saw blade by Penny (f. Penny-Dobroszyce sp. z o.o., Dobroszyce, Poland) [39]. These type of saw blades are dedicated and used in the re-sawing processes of birch (*Betula* L.), beech (*Fagus* sp.), hornbeam (*Carpinus betulus* L.), great maple (*Acer pseudoplatanus* L.), oak (*Quercus* L.), and ash (*Fraxinus* L.), as well as some exotic woods. These species are most frequently used in Poland for the production of the surface layers in composite flooring panels. Technical data of the saw blade were as follows: blade width B = 40 mm, saw blade thickness s = 0.9 mm, overall set (kerf) S_t_ = 1.4 mm, tooth pitch P = 15 mm, rake angle γ_f_ = 8°, wedge angle β_f_ = 71°, and clearance angle α_f_ = 11°. The macro dimensions of both saw blades were the same.

The regeneration of the narrow-kerf saw blade was conducted on the machine for automatic profile sharpening of gang saw blades controlled with 2 CNC axes in wet grinding GNP100 (f. Iseli + CO, AG, Maschinenfabrik, Schötz, Swiss) [40] by an external company providing tool sharpening services to sawmills and other woodworking plants. The sharpening process of the saw blades was carried out using grinding wheels EN12413 1 250 × 8 × 32 99BA 60 L 9 V30 40 m·s^−1^ (f. Tyrolit, Schwaz, Austria). The meanings of the symbols in this designation according to European standard EN12413 are as follows: 1 is the type of the grinding wheel, the outside diameter is 250 mm, the thickness of the wheel is 8 mm, the bore diameter is 32 mm, 99BA is abrasive material (in this case friable aluminum oxide), 60 is medium grit size, L is medium hardness of the grinding wheel, 9 is an open type of structure, V30 is vitrified bond, and 40 m·s^−1^ is the maximum rim speed.

**Figure 2:**
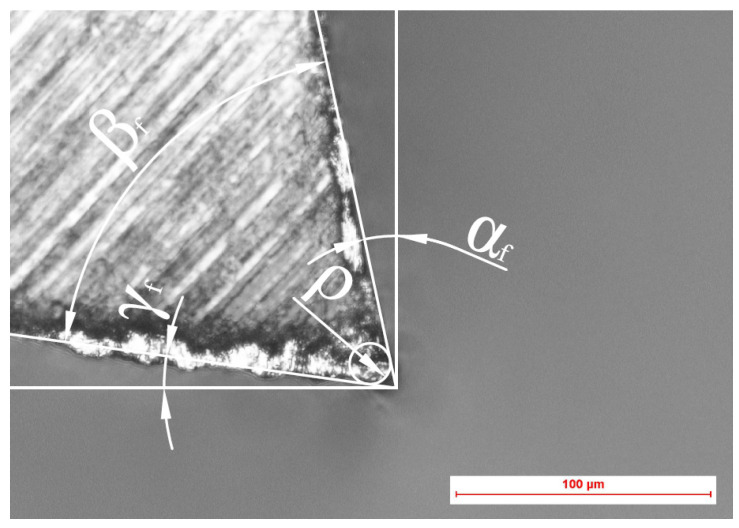
Methodology for measuring the cutting edge radius ρ based on a microscope image.

A NIKON ECLIPSE Ti-S microscope equipped with a NIKON DS-Fi2 recording camera was used to take pictures of each tooth. The camera is equipped with NIKON lenses with magnifications of 5×, 10×, 20×, and 50×. The obtained teeth images were additionally equipped with a scale. The images were imported to AutoCAD software (Autodesk Inc., San Rafael, CA, USA), where they were scaled according to the included scale and analysed to determine the values of the cutting edge radii (Figure 2).

The results of the obtained values of the cutting edges’ radii were subjected to statistical analyses. The first was Grubbs coarse errors analysis [41]. The second analysis performed on the results obtained was an analysis of variance (ANOVA), which was used to determine the significance of differences between the values of the cutting edges’ radii of the new saw blade and the sharpened saw blade [41].

## 3. Results and Discussion

**Figure 3:**
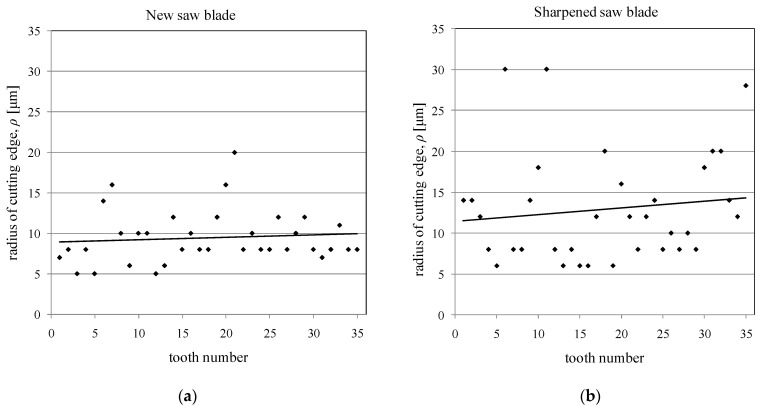
The radii of cutting edges determined from tooth images from both analysed saw blades: new saw blade (**a**) and sharpened saw blade (**b**).

**Figure 4:**
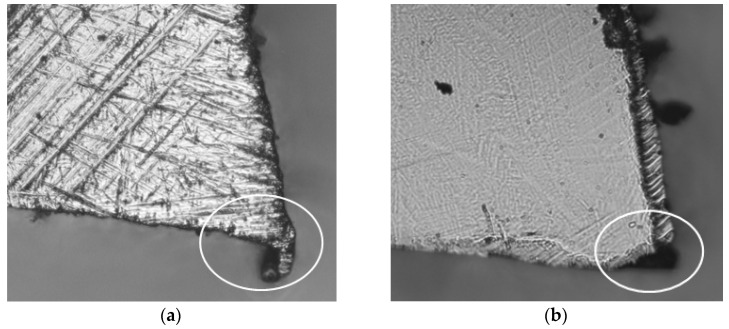
The teeth with larger values of the cutting edge radii, which are caused by burrs after grinding the cutting edge in a new saw blade (**a**) and by damage to the cutting edges in sharpened saw blades (**b**).

The obtained images from each tooth of both saw blades analysed were examined. The results of the analyses carried out to determine the cutting edge radii are presented in Figure 3. In Figure 3b, which shows the results for the sharpened saw blade, there are noticeable outliers from the overall point cloud. These much larger values of the cutting edge radii could be caused by excessive tooth wear during the preceding cutting process, tooth damage during saw blades installation and removal in gangs, or teeth damage during the sharpening process of saw blades. This damage, despite regenerative sharpening, causes a significant reduction in the sharpness of the cutting edge. An example of a tooth with a damaged cutting edge is shown in Figure 4b.

The larger cutting edge radii for the new blade (Figure 3a) were caused by the occurrence of burrs after the cutting edge grinding process (Figure 4a), which was the original sharpening process included in the blade manufacturing process. These burrs would probably have been removed during the initial stage of the sawing process, during the initial running-in period (the first part of the curve of natural wear) [42].

**Figure 5:**
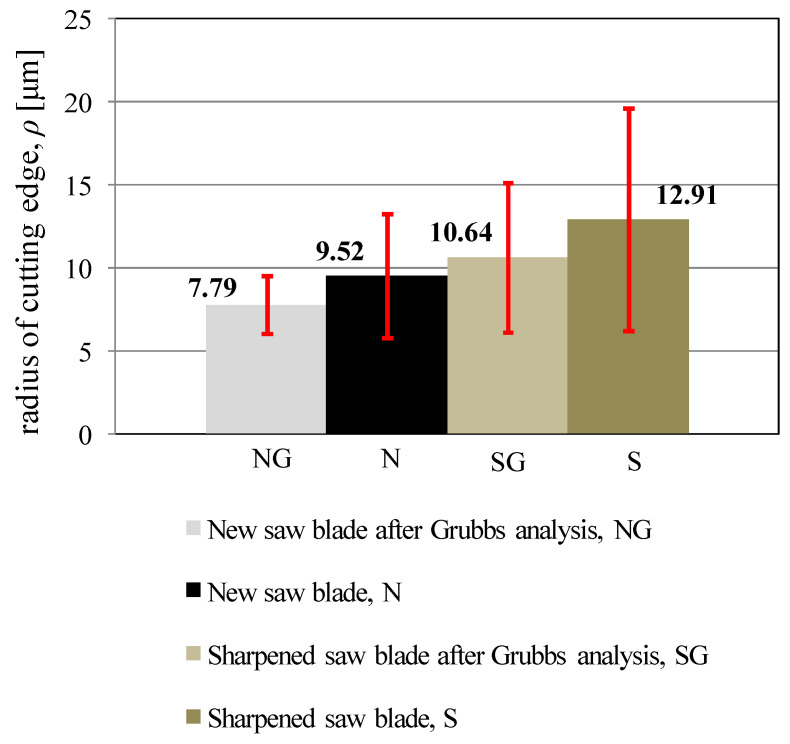
Mean values of the cutting edge radii with standard deviations.

Both cutting edge failures for a sharpened blade and cutting edge burrs for a new blade can be regarded as coarse errors in the measurement of the cutting edge radii. Therefore, these values were eliminated from the analysis using the Grubbs test [41]. The elimination of coarse errors noticeably affects the mean values of radii and standard deviations. The mean values of the cutting edge radii along with the standard deviations for both analysed blades, both before and after coarse error elimination, are shown in Figure 5.

The difference between the mean values of the cutting edge radii obtained for a new saw blade and a sharpened saw blade is noticeable in Figure 5. The values of standard deviations of the cutting edge radii are significantly smaller in the case of the new saw blade, which indicates a greater repeatability of the grinding process of teeth during manufacturing than sharpening process applied after cutting processes. Furthermore, according to Csanady and Magoss [3], the mean values of the cutting edge radii of both analysed saw blades are in the range, up to 20 μm, where the cutting edge is considered as sharp. However, only the mean radii of the cutting edges for a new saw blade are in the range (with standard deviations) where the wear stage of the cutting edge does not increase the value of cutting forces (up to 10 μm). Therefore, in the case of sharpened saw blades, it is necessary to choose cutting parameters for the sawing process that will take into account the possibility of generating higher values of cutting forces during the sawing process with these saw blades. These more important cutting parameters will be the feed speed, the number of saw blades in the gang, and the height of the material being cut.

Additionally, the statistical significance analysis of the differences between the values of the cutting edges’ radii of the new saw blade and the sharpened saw blade showed that these differences are statistically significant (Table 1). The statistical significance of the differences for the values of the cutting edges’ radii was demonstrated both for the values obtained before and after the Grubbs coarse errors analysis (Table 1). The statistical analysis carried out further confirms the fact that the cutting edges after the regenerative sharpening process are no longer as sharp as the cutting edges after sharpening in the manufacturing process. The observed phenomenon may lead to faster wear of the regenerated cutting blades as shown by Jaworski [8]. In this work [8], it was observed that as the number of sharpenings increases, the mean value of the pull broach tooth wear on the clearance face also increases, which has an impact on the quality of the machining process.

Csanady and Magoss [3] also proposed a relationship for determining the cutting edge radius based on the value of the edge angle and the measured value of the radial displacement of the tool corner KE. This relationship is right for the symmetrical shape of the worn edge.

After transformation, this relationship can take the form of Equation (1) and, based on the measured values of the cutting edge radii, can be used to determine the radial displacement of the tool corner, *KE*.

(1) $$KE=\rho \cdot \frac{1-\sin\left(\frac{\beta_{f}}{2}\right)}{\sin\left(\frac{\beta_{f}}{2}\right)}$$

**Figure 6:**
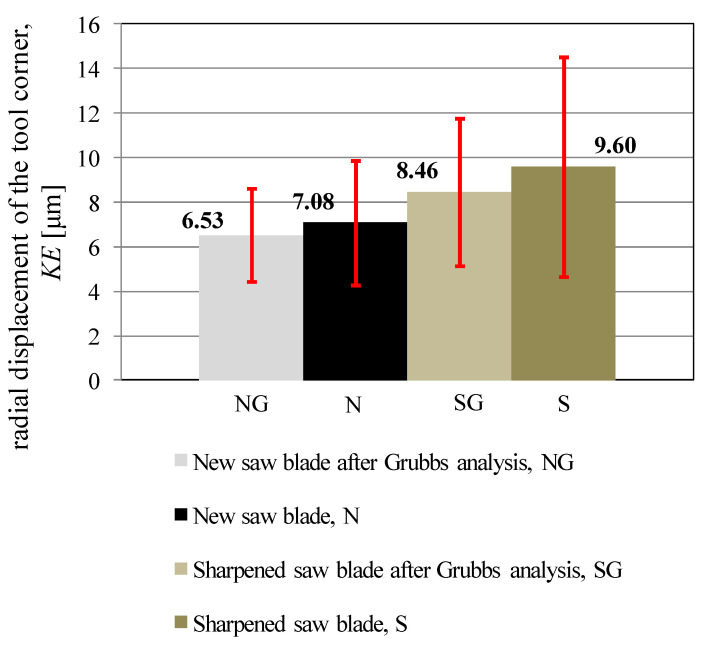
Mean values of the tool corner radial displacement KE with standard deviations.

The determined values of the radial displacement of the tool corner KE based on Equation (1) are shown in Figure 6. Comparing Figure 5 and Figure 6, it can be seen that the trends of changes in values are very similar, as well as the proportions of standard deviations. This may indicate that these two parameters describing the level of cutting edge wear may be interchangeable. This means that it is enough to measure only one of them to have information about the other one. Also, there is no justification for measuring both these parameters simultaneously.

## 4. Conclusions

The analysis carried out to determine the cutting edge radii has allowed the following conclusions:

- The mean values of the cutting edge radii of new saw blades (ρ_n_ = 7.79 μm) are smaller than the cutting edge radii of sharpened saw blades (ρ_s_ = 10.64 μm).
- The standard deviations for the mean value of the cutting edge radii of a new saw blade (SD_n_ = ±1.75 μm) are smaller than for the value obtained for sharpened saw blades (SD_s_ = ±4.47 μm), which indicates the greater repeatability of the primary grinding process of cutting edges over secondary sharpening.
- A statistically significant difference between the mean values of the cutting edge radii of a new saw blades and the cutting edge radii of sharpened saw blades was observed.
- Sharpened saw blades can generate higher values of cutting forces than new saw blades, which should be taken into account in the selection of sawing process parameters, especially the feed speed, the number of saw blades in the gang, and the height of the material being cut.

The direction of further research will be focused on an appropriate level of cutting blade wear that would ensure that the cutting blade can be regenerated to its original edge sharpness level.

## Figures and Tables

**Table 1:** Significance of differences of the cutting edge radii between a brand new and regenerated cutting saw blades (ANOVA) (α = 0.05).

**The Radii of Cutting Edges without Grubbs Analysis**							
**Sample Code**	**Source**	**DF**	**Adj SS**	**Adj MS**	**F-Value**	***p*-Value**	**F-Critical**
N	Between groups	1	194.485	194.485	6.864	0.0109	3.986
S	Within groups	66	1870.029	28.334			
	Total	67	2064.515				
**The Radii of Cutting Edges after Grubbs Analysis**							
NG	Between groups	1	75.777	75.777	4.936	0.0308	4.030
SG	Within groups	51	782.978	15.353			
	Total	52	858.755				

## References

[1] Prokofiev G.F., Tyurin A.M., Kabakova M.Y., Kovalenko O.I. (2020). Determination of the initial stiffness of unstretched rolled strip saws. Izvestiâ Vysših Učebnyh Zavedenij. Lesnoj Žurnal Russ. For. J..

[2] Orlowski K. (2003). Some approaches to the determination of saw blade stiffness. Drvna Ind..

[3] Csanady E., Magoss E. (2020). Mechanics of Wood Machining.

[4] Orlowski K.A., Sandak J., Chuchala D. (2020). Thickness accuracy of sash gang sawing. BioResources.

[5] Piotrowski A. (2015). Wpływ ostrzenia na dokładność frezów ślimakowych modułowych. (The impact of sharpening on accuracy of hobs). Mechanik.

[6] Stachurski W., Sawicki J., Krupanek K., Nadolny K. (2021). Application of numerical simulation to determine ability of air used in MQL method to clean grinding wheel active surface during sharpening of hob cutters. Int. J. Precis. Eng. Manuf.-Green Technol..

[7] Piotrowski A. (2018). Hob identification methods. Adv. Sci. Technol. Res. J..

[8] Jaworski J., Kluz K., Trzepieciński T. (2016). Wpływ ostrzenia na efektywność pracy przeciągacza (The effect of sharpening on efficiency of a pull broach operation). Mechanik.

[9] Klamecki B.E. (1979). A review of wood cutting tool wear literature. Holz Roh Werkst..

[10] Beer P. (2002). Obróbka Skrawaniem Obwodowym Drewna Nowo Opracowanymi Narzędziami. (Wood Peeling with New Elaborated Tools).

[11] Cięcie (2016). Poradnik Obróbki Skrawaniem.

[12] Svoreň J., Naščák L., Koleda P., Barcík Š., Němec M. (2021). The circular saw blade body modification by elastic material layer effecting circular saws sound pressure level when idling and cutting. Appl. Acoust..

[13] Igaz R., Kminiak R., Krišťák Ľ., Němec M., Gergeľ T. (2019). Methodology of Temperature Monitoring in the Process of CNC Machining of Solid Wood. Sustainability.

[14] Liu Y., Wang F., Lv J., Wang X. (2020). A Novel Method for Tool Identification and Wear Condition Assessment Based on Multi-Sensor Data. Appl. Sci..

[15] Twardowski P., Wiciak-Pikuła M. (2019). Prediction of Tool Wear Using Artificial Neural Networks during Turning of Hardened Steel. Materials.

[16] Altas E., Gokkaya H., Karatas M.A., Ozkan D. (2020). Analysis of Surface Roughness and Flank Wear Using the Taguchi Method in Milling of NiTi Shape Memory Alloy with Uncoated Tools. Coatings.

[17] Kuntoğlu M., Aslan A., Sağlam H., Pimenov D.Y., Giasin K., Mikolajczyk T. (2020). Optimization and Analysis of Surface Roughness, Flank Wear and 5 Different Sensorial Data via Tool Condition Monitoring System in Turning of AISI 5140. Sensors.

[18] Brili N., Ficko M., Klančnik S. (2021). Tool Condition Monitoring of the Cutting Capability of a Turning Tool Based on Thermography. Sensors.

[19] Denkena B., Krödel-Worbes A., Beblein S., Hein M. (2021). Influence of End Mill Manufacturing on Cutting Edge Quality and Wear Behavior. J. Manuf. Mater. Process..

[20] Zlamal T., Mrkvica I., Szotkowski T., Malotova S. (2019). The Influence of Surface Treatment of PVD Coating on Its Quality and Wear Resistant. Coatings.

[21] Orlicz T. (1988). Obróbka Drewna Narzędziami Tnącymi. (Wood Machining with Cutting Tools).

[22] Wasielewski R., Orlowski K. (2005). Inspection of circular saw teeth quality. Wood Res.-Slovak..

[23] Sandak J., Pałubicki B., Kowaluk G. Measurement of the cutting tool edge recession with optical methods. Proceedings of the 20th International Wood Machining Seminar.

[24] Palubicki B., Szulc M., Sandak J., Sinn G., Orlowski K.A. (2014). Method and device for 3D recognition of cutting edge micro geometry. Drvna Ind..

[25] Aquinas T. Ethical Conundrums. https://www.theguardian.com/notesandqueries/query/0,5753,-2029,00.html.

[26] Orlowski K.A., Ochrymiuk T., Hlaskova L., Chuchala D., Kopecky Z. (2020). Revisiting the estimation of cutting power with different energetic methods while sawing soft and hard woods on the circular sawing machine: A Central European case. Wood Sci. Technol..

[27] Sinn G., Chuchala D., Orlowski K.A., Taube P. (2020). Cutting model parameters from frame sawing of natural and impregnated Scots pine (*Pinus sylvestris* L.). Eur. J. Wood Wood Prod..

[28] Atkins A.G. (2009). The Science and Engineering of Cutting. The Mechanics and Process of Separating, Scratching and Puncturing Biomaterials, Metals and Non-Metals.

[29] Wang H., Chang L., Ye L., Williams J.G. Micro-cutting tests: A new way to measure the fracture toughness and yield stress of polymeric nanocomposites. Proceedings of the 13th International Conference on Fracture.

[30] Blackman B.R.K., Hoult T.R., Patel Y., Williams J.G. (2013). Tool sharpness as a factor in machining tests to determine toughness. Eng. Fract. Mech..

[31] Childs T.H.C. (2010). Surface energy, cutting edge radius and material flow stress size effects in continuous chip formation of metals. CIRP J. Manuf. Sci. Technol..

[32] Astakhov V.P. (2010). Geometry of Single-Point Turning Tools and Drills. Fundamentals and Practical Applications.

[33] Bariska M., Börcsök Z., Kantó Z., Czimondor D., Pásztory Z. (2016). Forces acting on saw teeth during timber processing—A practical approach. BioResources.

[34] Porankiewicz B., Sandak J., Tanaka C. (2005). Factors influencing steel tool wear when milling wood. Wood Sci. Technol..

[35] Cristóvão L., Lhate I., Grönlund A., Ekevad M., Sitoe R. (2011). Tool wear for lesser known tropical wood species. Wood Mater. Sci. Eng..

[36] Kminiak R., Gašparík M., Kvietková M. (2015). The dependence of surface quality on tool wear of circular saw blades during transversal sawing of beech wood. BioResources.

[37] Okai R., Tanaka C., Iwasaki Y. (2006). Influence of mechanical properties and mineral salts in wood species on tool wear of high-speed steels and stellite-tipped tools—Consideration of tool wear of the newly developed tip-inserted band saw. Holz Roh Werkst..

[38] https://www.wintersteiger.com/en/Woodtech/Saw-Blades-and-Service/Product-Range-Saws/Thin-cutting-saw-blades.

[39] https://penny.pl/pilki-z-cienkim-rzazem-do-minitrakow/.

[40] https://www.iseli-swiss.com/en/products/machines/gnp-100.

[41] Sachs L. (1984). Applied Statistics. A Handbook of Techniques.

[42] Grzesik W. (2017). Advanced Machining Processes of Metallic Materials: Theory, Modelling and Applications.

